# Familial immune‐mediated aplastic anaemia in six different families

**DOI:** 10.1002/jha2.722

**Published:** 2023-06-28

**Authors:** Tatsuya Imi, Hiroki Mizumaki, Kazuyoshi Hosomichi, Yasuhito Nannya, Yoshitaka Zaimoku, Takeshi Yoroidaka, Takamasa Katagiri, Ken Ishiyama, Hirohito Yamazaki, Ryosuke Ogawa, Mika Kuroiwa, Atsushi Tajima, Seishi Ogawa, Shinji Nakao

**Affiliations:** ^1^ Department of Hematology Graduate School of Medical Sciences Kanazawa University Kanazawa Japan; ^2^ Department of Bioinformatics and Genomics Graduate School of Advanced Preventive Medical Sciences Kanazawa University Kanazawa Japan; ^3^ Pathology and Tumor Biology Graduate School of Medicine Kyoto University Kyoto Japan; ^4^ Division of Hematopoietic Disease Control Institute of Medical Sciecen The University of Tokyo Tokyo Japan; ^5^ Department of Clinical Laboratory Sciences Graduate School of Medical Sciences Kanazawa University Kanazawa Japan; ^6^ Department of Hematology and Oncology Japan Community Health Care Organization Kyushu Hospital Fukuoka Japan; ^7^ Department of Hematology and Oncology National Hospital Organization Fukuoka Higashi Medical Center Fukuoka Japan

**Keywords:** aplastic anaemia, bone marrow failure, HLA, PNH

## Abstract

We studied the pathophysiology of aplastic anaemia (AA) in six different pairs of relatives without a family history of hematologic disorders or congenital AA. Five and four of the six pairs shared the HLA‐DRB1*15:01 and B*40:02 alleles, respectively. Glycosylphosphatidylinositol‐anchored protein‐deficient blood cells were detected in eight of the 10 patients evaluated. In a mother‐daughter pair from one family, flow cytometry detected leukocytes lacking HLA‐A2 due to loss of heterogeneity in chromosome 6p. Whole‐exome sequencing of the family pair revealed a missense mutation in *MYSM1*. These results suggest that genetic inheritance of immune traits might underlie familial AA in some patients.

**Abbreviations**
AA: aplastic anaemia
ALG: anti‐lymphocyte globulin
ATG: anti‐thymocyte globulin
CsA: cyclosporine
ESA: erythropoiesis‐stimulating agent
GPI: glycosylphosphatidylinisotol
HLA: human leukocyte antigen
mPSL: methylprednisolone
NA: not assessable
NSAA: non‐severe AA
PNH: paroxysmal nocturnal haemoglobinuria
PSL: prednisolone
rBMT: bone marrow transplantation from a related donor
SAA: severe AA

## 1 SHORT REPORT

While the occurrence of aplastic anaemia (AA) in multiple members of a family usually prompts suspicion of congenital AA (cAA), some patients with familial AA may inherit genetic susceptibility to immune‐mediated acquired AA (aAA) rather than germ‐line mutations linked to cAA [1]. From 1986 to 2016, we encountered six pairs of patients with AA from six different Japanese families (three mother and child pairs and three sibling pairs). AA was diagnosed according to the guidelines proposed by the British Society of Haematology while AA/paroxysmal nocturnal haemoglobinuria syndrome (AA/PNH) was diagnosed when the serum lactate dehydrogenase level exceeded 1.5 time above normal due to an increase in the percentage of glycosylphosphatidylinositol anchored protein‐deficient (glycosylphosphatidylinisotol (GPI)[‐]) cells in the peripheral blood of patients with AA. Bone marrow aspiration and biopsies were performed on all patients to confirm hypocellularity. Both of the two cases diagnosed with PNH did not have symptoms associated with hemolysis. In all cases, no family members besides the affected pairs had a history of AA. Table 1 and Table S1 summarizes the clinical characteristics, human leukocyte antigen (HLA) alleles, and results of GPI(‐) cell and HLA class I allele‐lacking (HLA[‐]) leukocyte screening of the 12 patients. Two pairs (Family 2 and Family 3) were diagnosed concurrently, while the other four pairs were diagnosed at intervals of 5 to 26 years. The two pairs who were diagnosed with AA at almost the same time did not share any signs of infections or exposure to the same medications or environmental toxins. None of the 12 patients had medical history of autoimmune diseases or any other diseases related to immune dysregulation. All 12 patients improved after treatment with cyclosporine A (CsA) and/or anti‐thymocyte globulin (*n* = 10), prednisolone (*n* = 1) or danazol (*n* = 1). All patients gave informed consent upon enrollment in observational studies on the presence of GPI(‐) blood cells and HLA(‐) leukocytes (see Supplemental Methods and Table S2). The study protocols were approved by the ethics committee of the Kanazawa University Institute of Medical, Pharmaceutical, and Health Sciences (numbers 876, 377 and 445).

**TABLE 1:** Clinical characteristics, HLA alleles and the presence of GPI(‐) blood cells and HLA(‐) leukocytes in 12 patients with familial AA.

Family number	Patient number	Relationship	Age at diagnosis	Year of diagnosis	Diagnosis	Treatment	HLA allele	GPI(‐) granulocytes	GPI(‐) erythrocytes	HLA‐lacking leukocytes
1	1	Mother	34	1987	PNH	PSL	A*2/24, B*35/60, DRB1*15:01/04:05	NA	NA	NA
	2	Son	7	1997	SAA	CsA, mPSL	A*2/2, B*35/61, DRB1*15:01/08:02	NA	NA	NA
2	3	Mother	43	1992	SAA	CsA, mPSL, mepitiostane	A*26/2, B*61/35, DRB1*15:01/08:02	+ (0.464%)	‐ (0.000%)	NA
	4	Daughter	5	1992	SAA	ALG, CsA, mPSL	A*26/24, B*61/51, DRB1*15:01/12:01	‐ (0.000%)*	‐ (0.000%)	NA
3	5	Elder sister	7	1986	SAA	ALG, mPSL, oxymetholone	A*31/33, B*61/44, DRB1*15:01/01:01	‐ (0.000%)*	+ (0.718%)	NA
	6	Younger sister	3	1986	SAA	ALG, mPSL, oxymetholone, mepitiostane	A*31/33, B*61/44, DRB1*15:01/01:01	‐ (0.000%)*	‐ (0.000%)	NA
4	7	Mother	26	1990	NSAA	danazol, ESA	A*02:06/24:02, B*55:02/40:02, C*01:02/03:04, DRB1*09:01/08:03	+ (0.271%)	+ (0.009%)	+ (65.7%)
	8	Daughter	25	2016	PNH	ATG, CsA	A*02:06/24:02, B*55:02/40:02, C*01:02/03:04, DRB1*09:01/08:02	+ (30.381%)	+ (10.065%)	+ (55.3%)
5	9	Elder sister	31	2002	NSAA	CsA	A*11:01/24:02, B*15:01/39:01, C*04:01/07:02, DRB1*11:01/15:01	+ (0.016%)	+ (0.005%)	‐
	10	Younger sister	34	2008	NSAA	CsA	A*11:01/24:02, B*15:01/39:01, C*04:01/07:02, DRB1*11:01/15:01	+ (0.231%)	+ (0.119%)	‐
6	11	Elder sister	56	2009	NSAA	CsA, rBMT	A*02:01/02:07, B*07:02/15:01, C*03:03/07:02, DRB1*01:01/15:01	+ (0.113%)*	+ (0.086%)	NA
	12	Younger brother	60	2014	SAA	CsA	A*02:01/02:07. B*07:02/15:01, C*03:03/07:02, DRB1*01:01/15:01	+ (9.133%)	+ (0.311%)	NA

*Note*: Clinical information and HLA typing, presence or absence of GPI‐AP(‐) and HLA(‐) blood cells of 12 patients are presented.

*indicates percentages of GPI(‐) granulocytes that were assessed using flow cytometry with anti‐CD55 and CD59 antibodies, rather than fluorescent‐labelled inactive toxin aerolysin (FLAER).

^+^indicates positive when data on the frequencies of GPI(‐) blood cells were unavailable.

**FIGURE 1:**
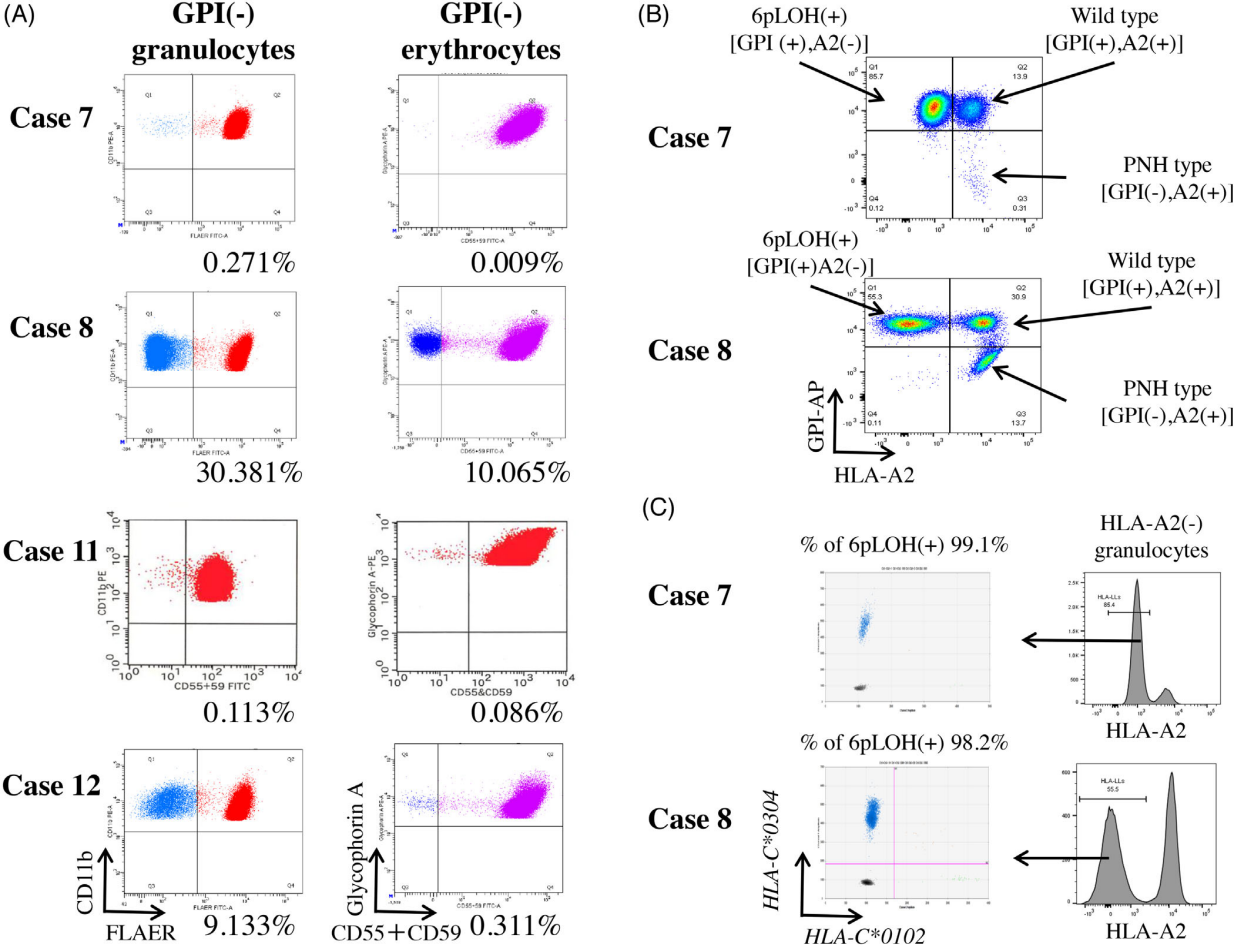
GPI(‐) blood cells and HLA(‐) leukocytes in two families. (A) GPI(‐) granulocytes and erythrocytes of four patients from two different families (cases 7, 8, 11 and 12). (B) HLA‐A2‐lacking cells in GPI(+) and GPI(‐)granulocytes of the mother (case 7) and daughter (case 8). (C) Percentages of 6pLOH(+) cells in sorted HLA‐A2‐lacking granulocytes of cases 7 and 8. The percentages were determined using HLA‐C alleles amplified by droplet digital PCR.

The HLA studies showed that five of the six pairs shared the HLA class II allele DRB1*15:01, the most frequently detected allele in AA patients, which is associated with the immune pathophysiology of AA and the development of PNH [2, 3]. Four pairs shared B61 (B*40:02), the allele that is most frequently lacking in AA patients due to copy neutral loss of heterozygosity of the 6p chromosome (6pLOH) or loss‐of‐function mutations [4, 5, 6]. Increased percentages of GPI(‐) granulocytes or erythrocytes were detected in eight (80%) of the 10 patients whose fresh blood samples were available for flow cytometry (FCM) (Figure 1A). Blood samples from five patients in three family pairs were analysed using FCM with anti‐HLA allelic antibodies (*n* = 4) to detect HLA(‐) leukocytes, and next‐generation sequencing of HLA class I regions (*n* = 4) and droplet digital PCR (ddPCR, *n* = 5) to detect 6pLOH or HLA allelic mutations [7]. Flow cytometry detected HLA(‐) leukocytes in two (cases 7 and 8 of family 4) of the five patients (Figure 1B,C). HLA‐A2‐lacking granulocytes accounted for 85.4% (case 7, mother) and 55.5% (case 8, daughter) of the total granulocytes and were exclusively detected in GPI(‐) granulocytes in both cases. The remaining three patients were negative for HLA(‐) leukocytes, as demonstrated by FCM, ddPCR and next‐generation sequencing.

We further investigated the reason underlying the lack of HLA‐A2 in leukocytes in cases 7 and 8. The ddPCR assay using probes against HLA‐C loci revealed that the percentage of 6pLOH‐positive cells in the sorted HLA‐A2‐lacking granulocytes was estimated to be 99.1% and 98.2% in cases 7 and 8, respectively (Figure 1C). The lost haplotype HLA‐A*02:06‐B*55:02‐C*01:02‐DRB1*09:01 was shared between these two patients. Case 8 had a healthy younger sister, who did not share this haplotype (Table S3). Targeted sequencing of 61 myeloid malignancy‐associated genes (Table S4) in HLA‐A2(‐) and HLA‐A2(+) granulocytes from case 8 showed a nonsense mutation (E115X) in exon 4 of *PIGA* with a variant allele frequency of 0.07 in HLA‐A2(+) granulocytes. No other somatic mutations were detected in either granulocyte subpopulation. To determine whether germline or somatic mutations in genes other than HLA class I alleles were shared by the members of Family 4 and Family 5, we performed whole‐exome sequencing of DNA from granulocytes, T cells and buccal cells from the four patients with an average sequencing depth of 245×, and found no pathological somatic mutations associated with hematological disorders (Table S5). However, a novel missense germline mutation of *MYSM1* was detected in family 4 (cases 7 and 8) during screening for recurrent germline mutations known to be associated with inherited bone marrow failure syndrome (Figure S1, Table S6). The *MYSM1* germline mutation was heterozygous, extremely rare with 0.0000 of minor allele frequencies, evaluated as uncertain significance by InterVar, and predicted to be a damaging or deleterious variant by three of six prediction scores according to Polyphen 2, likelihood ratio test and MutationTaster, whereas the other three scores, including SIFT, FATHMM and RadialSVM, predicted it to be tolerated or neutral. Copy number variation analysis of the sequencing data from the four patients confirmed the presence of 6pLOH in Cases 7 and 8 (Figure S2).

Based on laboratory findings, including the presence of immune markers (GPI[‐] cells and HLA[‐] leukocytes) and HLA alleles associated with susceptibility to AA (HLA‐DRB1*15:01 and HLA‐B*40:02), and a good response to immunosuppressive therapy, all 12 patients in the six different families were thought to have immune‐mediated bone marrow failure affected by genetic susceptibilities. The HLA‐A*02:06 allele contained in the lost HLA haplotype of the family 4 pair is one of the most frequently lost HLA class I alleles due to 6pLOH [8]. Family 4 also shared a *MYSM1* germline missense mutation. MYSM1 is a chromatin‐binding transcriptional cofactor that deubiquitinates histone H2A and is required for the maintenance of hematopoietic stem or progenitor cells, early lymphocyte development, and dendritic cell differentiation [9]. MYSM1 inhibits the pathogen recognition receptor pathways for pro‐inflammatory and type I interferon gene induction, acting as a key regulator of innate immunity [10, 11]. Several studies have demonstrated that *MYSM1* deficiency results in an autosomal recessive primary immunodeficiency associated with inherited bone marrow failure [12, 13]. Although the germline missense mutation of *MYSM1* detected in the members of family 4 occurred in only one haplotype, an excessive immune response to exogenous antigens due to haploinsufficiency of *MYSM1* may have increased susceptibility to the development of immune‐mediated AA in the two patients [14]. Given the lack of a shared HLA class I allele in the granulocytes of the two members of family 4, who lived together for a long time, it is tempting to speculate that a common environmental factor that led to the induction of cytotoxic T lymphocytes specific to common antigens presented by HLA‐A*02:06 may also have contributed to the development of AA [15].

In conclusion, the occurrence of familial AA does not always indicate congenital AA. Screening of laboratory markers, such as GPI(‐) cells and HLA(‐) leukocytes, is essential for the appropriate treatment of patients with familial AA.

## AUTHOR CONTRIBUTIONS

T.I. and H.M. contributed equally to this work. T.I., H.M. and S.N. designed the study and wrote the manuscript. T.I., H.M., K.I., H.Y., O.R., M.K. and S.N. collected clinical data and blood samples. T.I., H.M., T.Y., T.K. and Y.Z. analysed flow cytometry and droplet digital PCR data. T.I., K.H., T.A., Y.N. and S.O. performed deep sequencing and WES.

## CONFLICT OF INTEREST STATEMENT

The authors declare no conflict of interest associated with this study. The funders had no role in the study design, data collection and analysis, decision to publish or manuscript preparation.

## ETHICS STATEMENT

The study protocols were approved by the ethics committee of the Kanazawa University Institute of Medical, Pharmaceutical, and Health Sciences (Numbers 876, 377, and 445).

## PATIENT CONSENT STATEMENT

All patients gave informed consent upon enrollment in observational studies.

## Supporting information

Supporting Information

## Data Availability

The data that support the findings of this study are available from the corresponding author, S.N., upon reasonable request.
