# Supplementary material for: Familial immune‐mediated aplastic anaemia in six different families
Source: EJHaem. 2023 Jun 28;4(3):714–8. doi: 10.1002/jha2.722 (PMC10435714; doi:10.1002/jha2.722)
Supplement: Supplementary file 1 — Supporting Information [file JHA2-4-714-s001.docx]

**Supplemental information**

**Familial immune-mediated aplastic anemia in six different families**

Tatsuya Imi^1^, Hiroki Mizumaki^1^, Kazuyoshi Hosomichi^2^, Yasuhito Nannya^3,4^, Yoshitaka Zaimoku^1^, Takeshi Yoroidaka^1^, Takamasa Katagiri^5^, Ken Ishiyama^1^, Hirohito Yamazaki^1^, Ryosuke Ogawa^6^, Kuroiwa Mika^7^, Atsushi Tajima^2^, Seishi Ogawa^3^ and Shinji Nakao^1^

^1^Department of Hematology, Graduate School of Medical Sciences, Kanazawa University, Japan;

^2^Department of Bioinformatics and Genomics, Graduate School of Advanced Preventive Medical Sciences, Kanazawa University, Kanazawa, Japan;

^3^Pathology and Tumor Biology, Graduate School of Medicine, Kyoto University;

^4^Division of Hematopoietic Disease Control, Institute of Medical Sciecen, The University of Tokyo;

^5^Department of Clinical Laboratory Sciences, Graduate School of Medical Sciences, Kanazawa University, Japan;

^6^Department of Hematology and Oncology, Japan Community Health Care Organization Kyushu Hospital, Fukuoka, Japan;

^7^Department of Hematology and Oncology, National Hospital Organization Fukuoka Higashi Medical Center, Fukuoka, Koga, Japan.

Correspondence: Shinji Nakao, Department of Hematology, Graduate School of Medical Sciences, Kanazawa University, 13-1 Takaramachi, Kanazawa, Ishikawa 920-8640 Japan.

E-mail: [snakao8205@staff.kanazawa-u.ac.jp](mailto:snakao8205@staff.kanazawa-u.ac.jp).

**SUPPLEMENTAL METHODS**

Sample preparation for flowcytometry and cell sorting

Targeted deep sequencing

Whole-exome sequencing (WES)

The droplet digital PCR.

　 References

**SUPPLEMENTAL TABLES**

　Supplemental Table 1. Peripheral blood cell counts at diagnosis.

Supplemental Table 2. Monoclonal antibodies used for flow cytometry.

　Supplemental Table 3. HLA haplotypes of Family 4.

Supplemental Table 4. Genes analyzed with targeted sequencing.

　Supplemental Table 5. Somatic mutations detected by whole-exome sequencing.

　Supplemental Table 6. Inherited bone marrow failure-associated genes analyzed with whole-exome sequencing.

Supplemental Table 7. Germ line nonsynonymous mutations of the gene associated with inherited bone marrow failure syndromes in cases 7-10 identified by whole-exome sequencing.

**SUPPLEMENTAL FIGURES**

Supplemental Figure 1. A novel *MYSM1* germ-line mutation identified by whole-exome sequencing.

Supplemental Figure 2. Copy neutral loss of heterozygosity of the 6p chromosome of HLA-A allele lacking granulocytes validated by genomic copy number analysis.

SUPPLEMENTAL METHODS

***Sample preparation for flowcytometry and cell sorting***

Blood cells including erythrocytes, granulocytes were subjected to high sensitivity flow cytometry for detecting small populations of glycosylphosphatidylinositol anchored protein deficient (GPI[-]) cells. Fresh blood was diluted to 3% in phosphate-buffered saline (PBS), and then 50 µL was incubated with 7AAD, PE-labelled anti-glycophorin A and FITC-labelled anti-CD55 and anti-CD59 monoclonal antibodies (mAbs) at 4 degree for 30 minutes to detect GPI(-) erythrocytes. Leukocytes after erythrocytes lysis by a lysis buffer containing NH4Cl 8.26 g/L, KHCO3 1.0 g/L and EDTA-E4Na 0.037 g/L were also prepared for the detection of GPI(-) and HLA-A allele lacking (HLA[-]) leukocytes. After washing with PBS, 50 µL of the leukocyte suspension was incubated with FLAER or FITC-labelled anti-CD55 and anti-CD59 mAbs in combination with PE-labelled CD11b to detect GPI(-) granulocytes, or with anti-HLA-A allele-specific mAbs, including those specific to HLA-A2, A24 and A11, and mAbs specific for lineage markers including APC-labelled CD33, PerCP-Cy5.5-labelled CD3, APC-Cy7-labelled CD19 to detect HLA(-) leukocytes in granulocytes, monocytes, B cells and T cells. Data acquisition was performed after the sample preparation using a FACSCanto II instrument (BD Biosciences, Franklin Lakes, NJ, USA) and the data were analyzed using the FACSDiva software program. When the percentages of GPI(-) cells were more than 0.005% and 0.003% of erythrocytes and granulocytes, increased GPI(-) cells were judged to be positive. When HLA(-) leukocytes were detected, paired fractions including granulocytes that lacked the HLA-A allele and granulocytes that retained the HLA-A allele (HLA[+] granulocytes), as well as CD3^+^ T cells were sorted using a fluorescence-activated cell sorter (FACSAria™ Fusion, BD Biosciences). The sorted leukocyte populations were subjected to DNA extraction using a DNA extraction kit (Qiagen, Hilden, Germany). For Case 7, the sufficient numbers of HLA(+) granulocytes could not be obtained due to the overwhelming percentages of HLA(-) granulocytes, only HLA(-) granulocytes were analyzed, along with T cells and buccal mucosa cells.

***Targeted deep sequencing***

Sixty-one genes, which were previously found to be mutated in AA, PNH and MDS patients, were chosen for targeted capture using SeqCap EZ choice (Roche Diagnostics, Westfield, IN, USA; Supplemental Table 2).^1-4^ All DNA libraries of HLA(-) and HLA(+) granulocytes and T cells were prepared according to the manufacturer’s instructions. The captured targets were subjected to the paired-end sequencing using an Illumina MiSeq system (Illumina, San Diego, CA, USA). Paired-end 100-bp reads were mapped to the reference genome (GRCh37) using Burrows-Wheeler Aligner (bwa) version 0.7.12.^5^ bwa-generated SAM files were converted to the BAM format, then sorted and indexed using SAM tools v.1.2.^6^ Duplicated reads were marked with Picard v.1.52 (<https://github.com/broadinstitute/picard>). The heuristic somatic mutation caller, VarScan 2,^7^ was used for somatic mutation and LOH calling. The mutations were reviewed using Unified Genotyper in the Genome Analysis Toolkit (GATK) v3.4^8^ and the alignment data were visually compared among granulocytes, T cells and buccal mucosa cells using IGV.^9^ The functional information on somatic mutations was annotated using ANNOVAR.^10^ Mutations were validated by resequencing using MiSeq.

***Whole-exome sequencing (WES)***

WES was performed for the granulocytes, T cells and buccal mucosa cells of Case 7-10. WES libraries were generated using SureSelect Human All Exon v6 (Agilent Technologies), followed by sequencing of enriched fragments on NovaSeq 6000 (Illumina) with 150 bp paired-end mode with the target depth of 250. Mutation calling was performed using Genomon2 pipeline (URL: <https://genomon.readthedocs.io/ja/latest/>). Briefly, sequencing reads were aligned to the human genome reference (hg19) using bwa version 0.7.10 with default parameter settings. PCR duplicates were eliminated using picard-tools version 1.39 (http://picard.sourceforge.net/). Somatic mutations were detected by eliminating polymorphisms and sequencing errors. To achieve this, Genomon2 first discards any of low-quality, unreliable reads and variants, that are defined by the following criteria: (i) depth < 9, (ii) base call quality <20, (iii) mapping quality < 20. After further excluding those variants that are not supported by a sufficient number of reads (total reads ≥ 10 and variant reads ≥ 4), variant allele frequencies (VAFs) ≥ 0.02 (for tissue sample) and ≤ 0.1 (for normal control), the remaining variants are interrogated for the evidence that they are observed at significantly higher VAFs than expected for errors (P ≤ 10^-4^), where the significance is evaluated by EBCall algorithm, on the basis of an empirical VAF distribution as determined using WES data of non-paired peripheral blood samples from the same sequencing run (n = 15-20). Putative germ-line variants are also excluded by comparing VAFs with matched control using the Fisher’s test (≤ 10^-2^), which also eliminates remaining additional sequencing errors. All variants within repetitive sequences were excluded to obtain higher true positive rate at the expense of sensitivity. Non-synonymous germ-line mutations in the genes associated with inherited bone marrow failure, according previous reports^11,12^, were additionally screened.

***The droplet digital PCR***

To identify the presence of 6pLOH leukocytes in the whole blood or to calculate the percentages of 6pLOH(+) granulocytes in the sorted HLA(-) and HLA(+) granulocyte populations, DNA samples extracted from whole blood or sorted cells were subjected droplet digital PCR for the HLA-with a QX200 AutoDG Droplet Digital PCR System (Bio-Rad, Hercules, CA, USA) by comparing the copy number of each HLA allele as previously reported.^13^

**References**

1) Yoshizato T, Dumitriu B, Hosokawa K, et al. Somatic Mutations and Clonal Hematopoiesis in Aplastic Anemia. *N Engl J Med.* 2015; 373(1):35-47.

2) Kulasekararaj AG, Jiang J, Smith AE, et al. Somatic mutations identify a subgroup of aplastic anemia patients who progress to myelodysplastic syndrome. *Blood.* 2014; 124(17):2698-704.

3) Shen W, Clemente MJ, Hosono N, et al. Deep sequencing reveals stepwise mutation acquisition in paroxysmal nocturnal hemoglobinuria. *J Clin Invest.* 2014; 124(10):4529-38.

4) Heuser M, Schlarmann C, Dobbernack V, et al. Genetic characterization of acquired aplastic anemia by targeted sequencing. *Haematologica.* 2014; 99(9):e165-7.

5) Li H, Durbin R. Fast and accurate short read alignment with Burrows-Wheeler transform. Bioinformatics. 2009;25(14):1754-60.

6) Li H, Handsaker B, Wysoker A, Fennell T, et al. The Sequence Alignment/Map format and SAMtools. Bioinformatics. 2009;25(16):2078-9.

7) Koboldt DC, Zhang Q, Larson DE, et al. VarScan 2: somatic mutation and copy number alteration discovery in cancer by exome sequencing. Genome Res. 2012;22(3):568-76.

8) McKenna A, Hanna M, Banks E, et al. The Genome Analysis Toolkit: a MapReduce framework for analyzing next-generation DNA sequencing data. Genome Res. 2010;20(9):1297-303.

9) Robinson JT, Thorvaldsdóttir H, Winckler W, et al. Integrative genomics viewer. Nat Biotechnol. 2011;29(1):24-6.

10) Wang K, Li M, Hakonarson H. ANNOVAR: functional annotation of genetic variants from high-throughput sequencing data. Nucleic Acids Res. 2010;38(16):e164.

11) Shimamura, A, Alter, BP. Pathophysiology and management of inherited bone marrow failure syndromes. Blood Rev. 2010; 24(3):101-22.

12) Bluteau O, Sebert M, Leblanc T, et al. A landscape of germ line mutations in a cohort of inherited bone marrow failure patients. Blood. 2018 Feb 15;131(7):717-732.

13) Zaimoku Y, Takamatsu H, Hosomichi K, et al. Identification of an HLA class I allele closely involved in the auto-antigen presentation in acquired aplastic anemia. *Blood.*

SUPPLEMENTAL TABLES

**Supplemental Table 1. Peripheral blood cell counts at diagnosis.**

| **Family No** | **Patient No** | **Diagnosis** | White blood cells (×10^9^/L) | Neutrophils (×10^9^/L) | Hemoglobin (g/dL) | Platelets (×10^9^/L) | Reticulocytes  (×10^9^/L) |
| --- | --- | --- | --- | --- | --- | --- | --- |
| 1 | 1 | PNH | NA | NA | 8.0 | 100.0 | NA |
|  | 2 | SAA | 2.40 | 0.312 | 4.2 | 8.0 | 39 |
| 2 | 3 | SAA | NA | NA | NA | NA | NA |
|  | 4 | SAA | NA | NA | NA | NA | NA |
| 3 | 5 | SAA | NA | NA | NA | NA | NA |
|  | 6 | SAA | NA | NA | NA | NA | NA |
| 4 | 7 | NSAA | 2.30 | 1.265 | 8.6 | 56.0 | 23.0 |
|  | 8 | PNH | 1.40 | 0.924 | 5.6 | 35.0 | 33.0 |
| 5 | 9 | NSAA | NA | NA | NA | NA | NA |
|  | 10 | NSAA | 3.70 | 1.200 | 10.9 | 50.0 | 53.0 |
| 6 | 11 | NSAA | 6.30 | 2.950 | 10.0 | 15.0 | 67.0 |
|  | 12 | SAA | 2.40 | 0.624 | 5.7 | 4.0 | 34.0 |

Abbreviations:. PNH, paroxysmal nocturnal hemoglobinuria; AA, aplastic anemia; NSAA, non-severe AA; SAA, severe AA; NA, not available.

**Supplemental Table 2. Monoclonal antibodies used for flow cytometry.**

| Antigen | Isotype | Conjugate | Source |
| --- | --- | --- | --- |
| CD3 | Mouse IgG1 | PerCP-Cy5.5 | BD Biosciences |
| CD11b | Mouse IgG1 | PE | BD Biosciences |
| CD19 | Mouse IgG1 | APC-Cy7 | Beckman Coulter |
| CD33 | Mouse IgG1 | APC | Beckman Coulter |
| CD55 | Mouse IgG2a | FITC | BD Biosciences |
| CD59 | Mouse IgG2a | FITC | BD Biosciences |
| CD235a (Glycophorin A) | Mouse IgG1 | PE | Dako |
| HLA-A2/28 | Mouse IgG2a | FITC | One Lambda |
| HLA-A2/28 | Mouse IgG2a | PE | One Lambda |
| HLA-A9/24 | Mouse IgG2b | FITC | One Lambda |
| HLA-A11 | Mouse IgM | Biotin | Abcam |
| FLAER | NA | Alexa Flour® 488 | Cedarlane |
| Streptavidin | NA | PE | BD Biosciences |
| 7-AAD | NA | NA | BD Biosciences |

Abbreviations: FLAER, fluorescent-labelled inactive toxin aerolysin; 7-AAD, 7-Amino-Actinomycin D; NA, not applicable; PerCP-Cy5.5, peridinin-chlorophyll proteins-Cy5.5 tandem; PE, phycoerythrin; APC, allophycocyanin; APC-Cy7, allophycocyanin-Cy7 tandem; FITC, fluorescein isothiocyanate.

**Supplemental Table 3. HLA haplotypes of Family 4.**

| Family member | A | B | C | DRB1 | Haplotype |
| --- | --- | --- | --- | --- | --- |
| Mother (Case 7) | *A*0206* | *B*5502* | *C*0102* | *DRB1*0901* | a |
|  | *A*2402* | *B*4002* | *C*0304* | *DRB1*0803* | b |
| Father | *A*2402* | *B*4002* | *C*0304* | *DRB1*0802* | c |
|  | *-* | *B*5401* | *C*0102* | *DRB1*0405* | d |
| Daughter (Case 8) | *A*0206* | *B*5502* | *C*0102* | *DRB1*0901* | a |
|  | *A*2402* | *B*4002* | *C*0304* | *DRB1*0802* | c |
| Younger sister | *A*2402* | *B*4002* | *C*0304* | *DRB1*0803* | b |
|  | *-* | *-* | *-* | *DRB1*0802* | c |

DNA samples from four family members in Family 4 were subjected to HLA allele typing. The mother (Case 7) and daughter (Case 8) share one haplotype.

**Supplemental Table 4. Genes analyzed with targeted sequencing.**

| *ASXL1* | *TERT* | *SMC3* | *RFX1* |
| --- | --- | --- | --- |
| *ATRX* | *SRSF2* | *BRCC3* |  |
| *DNMT3A* | *U2AF1* | *CBL* |  |
| *EED* | *ZRSR2* | *DIS3* |  |
| *EZH2* | *RAD21* | *RIT1* |  |
| *TET2* | *STAG2* | *PRPF8* |  |
| *ATM* | *LAMB4* | *DHX29* |  |
| *BCOR* | *PEG3* | *MECOM* |  |
| *BCORL1* | *PIGA* | *KDM6A* |  |
| *PHF6* | *SETBP1* | *ETV6* |  |
| *RUNX1* | *IDH2* | *BRPF1* |  |
| *TP53* | *RBBP4* | *CCR9* |  |
| *WT1* | *SUZ12* | *KDM3B* |  |
| *GNAS* | *CUX1* | *C11orf34 (PLET1)* |  |
| *KRAS* | *CSMD1* | *IKZF1* |  |
| *NF1* | *JAK1* | *ERBB2* |  |
| *STAT3* | *JAK2* | *SLIT1* |  |
| *MPL* | *JAK3* | *LRCH1* |  |
| *POT1* | *SH2B3* | *DPP4* |  |
| *RAP1* | *SMC1A* | *PRR5L* |  |

**Supplemental Table 5. Somatic mutations detected by whole-exome sequencing.**

| Case | Sample | Gene | Chromosome | Exon | Exonic Function | Genome change | Amino acid change | VAF | dbSNP138 | COSMIC ID |
| --- | --- | --- | --- | --- | --- | --- | --- | --- | --- | --- |
| 7 | 6pLOH granulocytes | CC2D1B | 1 | 20 | nonsynonymous SNV | G2158A | D720N | 0.396 | rs200283353 | COSM910638 |
| 7 | 6pLOH granulocytes | MGMT | 10 | 5 | nonsynonymous SNV | C616T | R206W | 0.133 | rs376927510 | None |
| 7 | 6pLOH granulocytes | CLPX | 15 | 12 | stopgain | C1669T | R557X | 0.133 | None | COSM1374079 |
| 7 | 6pLOH granulocytes | GPC4 | X | 9 | nonsynonymous SNV | A1477G | S493G | 0.149 | None | None |
| 7 | T cells | PLXNB3 | X | 30 | nonsynonymous SNV | G5033A | R1678Q | 0.109 | None | COSM3780390, COSM3780389 |
| 8 | 6pLOH granulocytes | ZSWIM3 | 20 | 2 | nonsynonymous SNV | C1928T | S643F | 0.107 | None | None |
| 8 | T cells | FBLN2 | 3 | 2 | nonsynonymous SNV | C1076T | A359V | 0.179 | None | None |
| 8 | T cells | COL6A6 | 3 | 5 | nonsynonymous SNV | G2172T | K724N | 0.228 | None | None |
| 9 | granulocytes | TFB2M | 1 | 1 | nonsynonymous SNV | G19T | G7W | 0.113 | None | None |
| 9 | granulocytes | PBRM1 | 3 | 3 | stopgain | C232T | R78X | 0.171 | None | None |
| 9 | granulocytes | PCDHA8 | 5 | 1 | nonsynonymous SNV | T395C | V132A | 0.108 | None | None |
| 9 | granulocytes | PIGO | 9 | 7 | stopgain | T2435G | L812X | 0.155 | None | None |
| 9 | granulocytes | CLDN9 | 16 | 1 | nonsynonymous SNV | A470T | K157M | 0.109 | None | None |
| 9 | T cells | RBFOX1 | 16 | 3 | nonsynonymous SNV | C412T | R138W | 0.136 | None | COSM435783, COSM435784, COSM1134040 |
| 10 | granulocytes | CORO1A | 16 | 5 | nonsynonymous SNV | C586T | R196C | 0.102 | None | None |
| 10 | T cells | MCM7 | 7 | 11 | nonsynonymous SNV | A1147C | M383L | 0.125 | None | None |

**Supplemental Table 6. Inherited bone marrow failure-associated genes analyzed with whole-exome sequencing.**

| ABL1 | DKC1 | ITGA2B | PTPN11 | RUNX1 | ZFPM1 |
| --- | --- | --- | --- | --- | --- |
| ABL2 | DNAJC21 | KIT | RAD51/FANCR | SALL4 |  |
| ACD | ENG | LIG4 | RAD51C/FANCO | SAMD9 |  |
| ALAS2 | ERCC4/FANCQ | MASTL/ACBD5 | RBM8A | SAMD9L |  |
| ASXL1 | ERCC6L2 | MDM2 | REV7/FANCV | SBDS |  |
| ATR | ERG | MDM4 | RPL11 | SH2B3 |  |
| BCL2L10 | ETS2 | MECOM | RPL15 | SLC37A4 |  |
| BRCA1/FANCS | ETV6 | MKRN1 | RPL23 | SLX4/FANCP |  |
| BRCA2/FANCD1 | FANCB | MYH9 | RPL27 | SRP72 |  |
| CBL | FANCE | MYSM1 | RPL31 | STIM1 |  |
| CDAN1 | FANCF | NBEAL2 | RPL35A | TAL1 |  |
| CEBPA | FANCI | NHP2 | RPL36 | TERC |  |
| CFHR1 | FANCL | NOP10 | RPL5 | TERT |  |
| CFHR3 | GATA1 | PALB2/FANCN | RPS10 | TINF2 |  |
| CFI | GATA2 | PARN | RPS17 | TNFRSF13B |  |
| CHEK2 | GFI1 | PAX5 | RPS24 | TP53 |  |
| CLCN7 | GP1BA | PIEZO1 | RPS28 | TP53BP2 |  |
| CTC1 | GP1BA | POT1 | RPS7 | UBE2T/FANCT |  |
| CYCS | GP1BB | PPM1D | RPS72A | WRAP53 |  |
| DDX41 | HOXA11 | PRF1 | RTEL1 | XRCC2/FANCU |  |

**Supplemental Table 7. Germ line nonsynonymous mutations of the gene associated with inherited bone marrow failure syndromes in cases 7-10 identified by whole-exome sequencing.**

| Case | Sample | Gene | Chromosome | Exon | ExonicFunc | Genome change | Amino acid change | Zygosity | dbSNP138 | COSMIC ID | InterVar | MAF |
| --- | --- | --- | --- | --- | --- | --- | --- | --- | --- | --- | --- | --- |
| 7 | buccal/T cell | MYSM1 | 1 | 8 | nonsynonymous SNV | A1166T | E389V | hetero | Novel | None | Uncertain significance | 0.0000 |
| 7 | buccal/T cell | RIT1 | 1 | 2 | nonsynonymous SNV | G31C | E11Q | hetero | rs493446 | None | Benign | 0.8174 |
| 7 | buccal/T cell | EPCAM | 2 | 3 | nonsynonymous SNV | T344C | M115T | homo | rs1126497 | None | Benign | 0.6033 |
| 7 | buccal/T cell | WIPF1 | 2 | 5 | nonsynonymous SNV | C593T | P198L | homo | rs4972450 | None | Benign | 0.9292 |
| 7 | buccal/T cell | NBEAL2 | 3 | 13 | nonsynonymous SNV | G1340A | R447H | hetoro | rs17079425 | None | Benign | 0.0349 |
| 7 | buccal/T cell | NBEAL2 | 3 | 37 | nonsynonymous SNV | C6161T | S2054F | hetero | rs2305637 | None | Benign | 0.145 |
| 7 | buccal/T cell | GATA2 | 3 | 3 | nonsynonymous SNV | G490A | A164T | hetero | rs2335052 | COSM445531 | Benign | 0.18 |
| 7 | buccal/T cell | ATR | 3 | 4 | nonsynonymous SNV | T632C | M211T | hetero | rs2227928 | COSM149487 | Benign | 0.6291 |
| 7 | buccal/T cell | WDR1 | 4 | 5 | nonsynonymous SNV | A553G | I185V | homo | rs13441 | None | Benign | 0.6196 |
| 7 | buccal/T cell | SRP72 | 4 | 1 | nonsynonymous SNV | G19T | G7W | hetero | rs17524437 | None | Likely benign | 0.004 |
| 7 | buccal/T cell | CFI | 4 | 11 | nonsynonymous SNV | G1217A | R406H | hetero | rs74817407 | COSM149725 | Likely benign | 0.0102 |
| 7 | buccal/T cell | PGM3 | 6 | 11 | nonsynonymous SNV | G1153A | D385N | hetero | rs473267 | None | Benign | 0.3463 |
| 7 | buccal/T cell | IKZF1 | 7 | 4 | nonsynonymous SNV | A490G | R164G | hetero | rs10899750 | None | Benign | 0.7485 |
| 7 | buccal/T cell | SAMD9 | 7 | 2 | nonsynonymous SNV | C1436T | T479M | hetero | rs78564070 | None | Benign | 0.0502 |
| 7 | buccal/T cell | SAMD9 | 7 | 2 | nonsynonymous SNV | G1360A | A454T | hetero | rs117649834 | None | Benign | 0.0305 |
| 7 | buccal/T cell | SAMD9L | 7 | 4 | nonsynonymous SNV | G796A | V266I | hetero | rs10488532 | None | Benign | 0.0868 |
| 7 | buccal/T cell | VPS13B | 8 | 8 | stopgain | T1239G | Y413X | homo | rs7460625 | None | Benign | 0.7491 |
| 7 | buccal/T cell | PAX5 | 9 | 7 | nonsynonymous SNV | C791T | T264I | homo | rs3780135 | COSM4163845 | Benign | 0.7818 |
| 7 | buccal/T cell | ERCC6L2 | 9 | 1 | nonsynonymous SNV | A1G | M1V | homo | rs690528 | COSM456305 | Benign | 0.2665 |
| 7 | buccal/T cell | ANKRD26 | 10 | 1 | nonsynonymous SNV | A59G | Q20R | homo | rs7897309 | None | Benign | 0.9039 |
| 7 | buccal/T cell | MASTL | 10 | 8 | nonsynonymous SNV | C1858G | P620A | hetero | rs3802526 | None | Benign | 0.0842 |
| 8 | buccal/T cell | MYSM1 | 1 | 8 | nonsynonymous SNV | A1166T | E389V | hetero | Novel | None | Uncertain significance | 0.0000 |
| 8 | buccal/T cell | MYSM1 | 1 | 8 | nonsynonymous SNV | A790G | T264A | hetero | rs12139511 | None | Benign | 0.6411 |
| 8 | buccal/T cell | RIT1 | 1 | 2 | nonsynonymous SNV | G31C | E11Q | homo | rs493446 | None | Benign | 0.8174 |
| 8 | buccal/T cell | EPCAM | 2 | 3 | nonsynonymous SNV | T344C | M115T | homo | rs1126497 | None | Benign | 0.6033 |
| 8 | buccal/T cell | EPCAM | 2 | 5 | nonsynonymous SNV | C515T | T172M | hetero | rs74531854 | COSM148851 | Benign | 0.0043 |
| 8 | buccal/T cell | WIPF1 | 2 | 5 | nonsynonymous SNV | C593T | P198L | homo | rs4972450 | None | Benign | 0.9292 |
| 8 | buccal/T cell | NBEAL2 | 3 | 13 | nonsynonymous SNV | G1340A | R447H | hetero | rs17079425 | None | Benign | 0.0349 |
| 8 | buccal/T cell | NBEAL2 | 3 | 37 | nonsynonymous SNV | C6161T | S2054F | hetero | rs2305637 | None | Benign | 0.145 |
| 8 | buccal/T cell | GATA2 | 3 | 3 | nonsynonymous SNV | C748G | P250A | hetero | rs78245253 | None | Benign | 0.0033 |
| 8 | buccal/T cell | ATR | 3 | 4 | nonsynonymous SNV | T632C | M211T | hetero | rs2227928 | COSM149487 | Benign | 0.6291 |
| 8 | buccal/T cell | WDR1 | 4 | 5 | nonsynonymous SNV | A553G | I185V | homo | rs13441 | None | Benign | 0.6196 |
| 8 | buccal/T cell | KIT | 4 | 10 | nonsynonymous SNV | A1621C | M541L | hetero | rs3822214 | COSM28026 | Benign | 0.0709 |
| 8 | buccal/T cell | SRP72 | 4 | 1 | nonsynonymous SNV | G19T | G7W | hetero | rs17524437 | None | Likely benign | 0.004 |
| 8 | buccal/T cell | IKZF1 | 7 | 4 | nonsynonymous SNV | A490G | R164G | homo | rs10899750 | None | Benign | 0.7485 |
| 8 | buccal/T cell | SAMD9 | 7 | 2 | nonsynonymous SNV | G1645T | V549L | hetero | rs10279499 | None | Benign | 0.0993 |
| 8 | buccal/T cell | SAMD9 | 7 | 2 | nonsynonymous SNV | C1436T | T479M | hetero | rs78564070 | None | Benign | 0.0502 |
| 8 | buccal/T cell | SAMD9 | 7 | 2 | nonsynonymous SNV | G1360A | A454T | hetero | rs117649834 | None | Benign | 0.0305 |
| 8 | buccal/T cell | SAMD9 | 7 | 2 | nonsynonymous SNV | T428C | I143T | hetero | rs6969691 | None | Benign | 0.1538 |
| 8 | buccal/T cell | SAMD9L | 7 | 4 | nonsynonymous SNV | A4547C | N1516T | hetero | rs10282508 | None | Benign | 0.0918 |
| 8 | buccal/T cell | SAMD9L | 7 | 4 | nonsynonymous SNV | G796A | V266I | hetero | rs10488532 | None | Benign | 0.0868 |
| 8 | buccal/T cell | MKRN1 | 7 | 4 | nonsynonymous SNV | G727C | V243L | hetero | rs2272095 | None | Benign | 0.2008 |
| 8 | buccal/T cell | VPS13B | 8 | 8 | stopgain | T1239G | Y413X | hetero | rs7460625 | None | Benign | 0.7491 |
| 8 | buccal/T cell | PAX5 | 9 | 7 | nonsynonymous SNV | C791T | T264I | homo | rs3780135 | COSM4163845 | Benign | 0.7818 |
| 8 | buccal/T cell | ERCC6L2 | 9 | 1 | nonsynonymous SNV | A1G | M1V | hetero | rs690528 | COSM456305 | Benign | 0.2665 |
| 8 | buccal/T cell | ERCC6L2 | 9 | 16 | nonsynonymous SNV | A2891T | N964I | hetero | rs3780574 | None | Benign | 0.1298 |
| 8 | buccal/T cell | ANKRD26 | 10 | 1 | nonsynonymous SNV | A59G | Q20R | homo | rs7897309 | None | Benign | 0.9039 |
| 8 | buccal/T cell | MASTL | 10 | 8 | nonsynonymous SNV | C1858G | P620A | hetero | rs3802526 | None | Benign | 0.0842 |
| 9 | buccal/T cell | MYSM1 | 1 | 8 | nonsynonymous SNV | A790G | T264A | hetero | rs12139511 | None | Benign | 0.6411 |
| 9 | buccal/T cell | MYSM1 | 1 | 8 | nonsynonymous SNV | T598A | C200S | hetero | rs17118103 | COSM146528 | Benign | 0.1612 |
| 9 | buccal/T cell | RIT1 | 1 | 2 | nonsynonymous SNV | G31C | E11Q | homo | rs493446 | None | Benign | 0.8174 |
| 9 | buccal/T cell | EPCAM | 2 | 3 | nonsynonymous SNV | T344C | M115T | homo | rs1126497 | None | Benign | 0.6033 |
| 9 | buccal/T cell | WIPF1 | 2 | 5 | nonsynonymous SNV | C593T | P198L | homo | rs4972450 | None | Benign | 0.9292 |
| 9 | buccal/T cell | NBEAL2 | 3 | 37 | nonsynonymous SNV | C6161T | S2054F | homo | rs2305637 | None | Benign | 0.0349 |
| 9 | buccal/T cell | GATA2 | 3 | 3 | nonsynonymous SNV | C748G | P250A | homo | rs78245253 | None | Benign | 0.0033 |
| 9 | buccal/T cell | CFI | 4 | 11 | nonsynonymous SNV | G1217A | R406H | hetero | rs74817407 | COSM149725 | Likely benign | 0.0102 |
| 9 | buccal/T cell | IKZF1 | 7 | 4 | nonsynonymous SNV | A490G | R164G | hetero | rs10899750 | None | Benign | 0.7485 |
| 9 | buccal/T cell | VPS13B | 8 | 8 | stopgain | T1239G | Y413X | homo | rs7460625 | None | Benign | 0.7491 |
| 9 | buccal/T cell | CYC1 | 8 | 2 | nonsynonymous SNV | A226G | M76V | homo | rs7820984 | None | Benign | 0.9623 |
| 9 | buccal/T cell | PAX5 | 9 | 7 | nonsynonymous SNV | C791T | T264I | homo | rs3780135 | COSM4163845 | Benign | 0.7818 |
| 9 | buccal/T cell | ERCC6L2 | 9 | 11 | nonsynonymous SNV | T1775C | V592A | homo | rs2274654 | None | Benign | 0.1758 |
| 9 | buccal/T cell | ERCC6L2 | 9 | 16 | nonsynonymous SNV | A2891T | N964I | homo | rs3780574 | None | Benign | 0.1298 |
| 9 | buccal/T cell | ENG | 9 | 8 | nonsynonymous SNV | G1096C | D366H | hetero | rs1800956 | None | Benign | 0.0069 |
| 9 | buccal/T cell | ANKRD26 | 10 | 1 | nonsynonymous SNV | A59G | Q20R | homo | rs7897309 | None | Benign | 0.9039 |
| 9 | buccal/T cell | MASTL | 10 | 8 | nonsynonymous SNV | C1010A | T337K | hetero | rs36121140 | None | Benign | 0.0074 |
| 10 | buccal/T cell | MYSM1 | 1 | 8 | nonsynonymous SNV | A790G | T264A | hetero | rs12139511 | None | Benign | 0.6411 |
| 10 | buccal/T cell | MYSM1 | 1 | 8 | nonsynonymous SNV | T598A | C200S | hetero | rs17118103 | COSM146528 | Benign | 0.1612 |
| 10 | buccal/T cell | RIT1 | 1 | 2 | nonsynonymous SNV | G31C | E11Q | homo | rs493446 | None | Benign | 0.8174 |
| 10 | buccal/T cell | EPCAM | 2 | 3 | nonsynonymous SNV | T344C | M115T | homo | rs1126497 | None | Benign | 0.6033 |
| 10 | buccal/T cell | WIPF1 | 2 | 5 | nonsynonymous SNV | C593T | P198L | homo | rs4972450 | None | Benign | 0.9292 |
| 10 | buccal/T cell | NBEAL2 | 3 | 37 | nonsynonymous SNV | C6161T | S2054F | homo | rs2305637 | None | Benign | 0.0349 |
| 10 | buccal/T cell | GATA2 | 3 | 3 | nonsynonymous SNV | C748G | P250A | hetero | rs78245253 | None | Benign | 0.0033 |
| 10 | buccal/T cell | GATA2 | 3 | 3 | nonsynonymous SNV | G490A | A164T | hetero | rs2335052 | COSM445531 | Benign | 0.18 |
| 10 | buccal/T cell | WDR1 | 4 | 5 | nonsynonymous SNV | A553G | I185V | hetero | rs13441 | None | Benign | 0.6196 |
| 10 | buccal/T cell | CFI | 4 | 11 | nonsynonymous SNV | G1217A | R406H | hetero | rs74817407 | COSM149725 | Likely benign | 0.0102 |
| 10 | buccal/T cell | PGM3 | 6 | 11 | nonsynonymous SNV | G1153A | D385N | hetero | rs473267 | None | Benign | 0.3463 |
| 10 | buccal/T cell | IKZF1 | 7 | 4 | nonsynonymous SNV | A490G | R164G | homo | rs10899750 | None | Benign | 0.7485 |
| 10 | buccal/T cell | SAMD9L | 7 | 4 | nonsynonymous SNV | T866C | F289S | hetero | rs2073793 | None | Uncertain significance | 0.0124 |
| 10 | buccal/T cell | VPS13B | 8 | 8 | stopgain | T1239G | Y413X | hetero | rs7460625 | None | Benign | 0.7491 |
| 10 | buccal/T cell | CYC1 | 8 | 2 | nonsynonymous SNV | A226G | M76V | homo | rs7820984 | None | Benign | 0.9623 |
| 10 | buccal/T cell | PAX5 | 9 | 7 | nonsynonymous SNV | C791T | T264I | homo | rs3780135 | COSM4163845 | Benign | 0.7818 |
| 10 | buccal/T cell | ERCC6L2 | 9 | 16 | nonsynonymous SNV | A2891T | N964I | homo | rs3780574 | None | Benign | 0.1298 |
| 10 | buccal/T cell | ENG | 9 | 8 | nonsynonymous SNV | G1096C | D366H | hetero | rs1800956 | None | Benign | 0.0069 |
| 10 | buccal/T cell | ANKRD26 | 10 | 25 | nonsynonymous SNV | G3655C | V1219L | hetero | rs12572862 | None | Benign | 0.0191 |
| 10 | buccal/T cell | ANKRD26 | 10 | 1 | nonsynonymous SNV | A59G | Q20R | homo | rs7897309 | None | Benign | 0.9039 |
| 10 | buccal/T cell | MASTL | 10 | 8 | nonsynonymous SNV | C1858G | P620A | hetero | rs3802526 | None | Benign | 0.0842 |

SUPPLEMENTAL FIGURES

**Supplemental Figure 1. A novel *MYSM1* germ-line mutation was identified by whole-exome sequencing.**


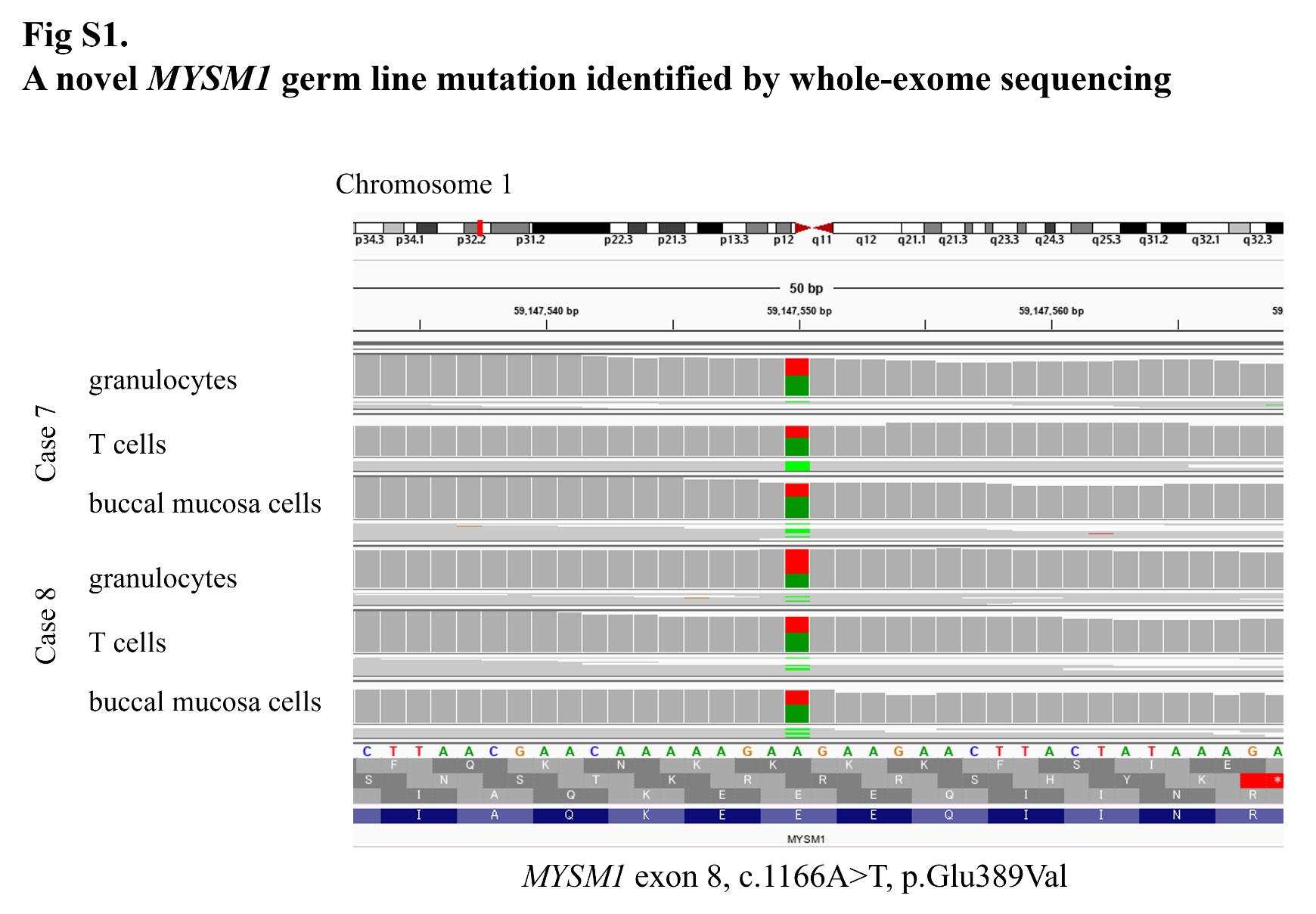


Whole- exome sequencing of Case 7 and 8’s granulocytes, T cells and buccal mucosa cells revealed a novel heterogeneous germ line missense mutation in exon 8 of *MYSM1* gene.

**Supplemental Figure 2. Copy neutral loss of heterozygosity of the 6p chromosome of HLA-A allele-lacking granulocytes validated by genomic copy number analysis.**


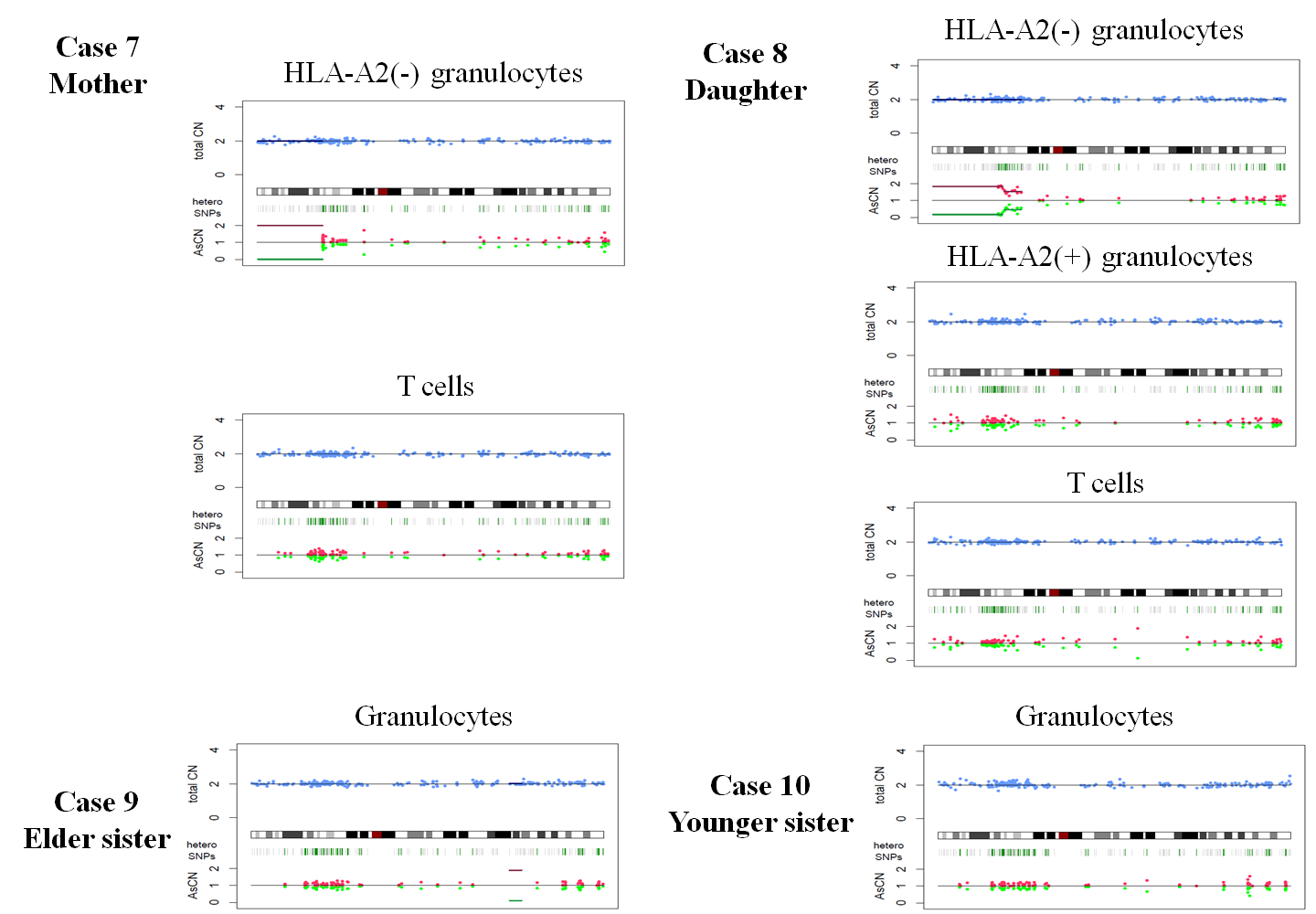


Genomic copy number analysis was performed by integrated data from single nucleotide polymorphisms obtained from whole-exome sequencing. The genomic copy number analyses in chromosome 6 of Case 7-10 are shown. 6pLOH were detected in HLA-A allele-lacking granulocytes of Case 7 and 8.
